# A multimodal approach connecting cortical and behavioural responses to the visual continuity illusion

**DOI:** 10.1177/23982128241251685

**Published:** 2024-05-06

**Authors:** Matthew C. D. Bailey, Johann F. du Hoffmann, Jeffrey W. Dalley

**Affiliations:** 1 Department of Psychology, University of Cambridge, Cambridge, UK; 2 Boehringer Ingelheim Pharma GmbH & Co, Biberach an der Riss, Germany; 3 Department of Psychiatry, Herchel Smith Building for Brain and Mind Sciences, University of Cambridge, Cambridge, UK

**Keywords:** Behaviour, mutli-modal approaches, EEG, fMRI, superior colliculus, visual perception, flicker fusion frequency, neurometric correlation

## Abstract

In their recently published study, Gil, Valente and Shemesh combined behaviour, functional magnetic resonance imaging, electroencephalography and causal interventions to establish and validate a cortical processing substrate underlying the transition from static to dynamic visual states in the rat. Their research highlights the superior colliculus as the primary mediator of visual temporal discrimination by showing a direct correlation between behavioural and cortically derived flicker fusion frequency thresholds. This work provides the first empirical evidence addressing the previously established disparity between behavioural and cortically derived flicker fusion frequency thresholds. It demonstrates how important convergent multimodal approaches are to mapping and validating previously disputed cortical pathways. Here, we discuss and evaluate their work, suggesting possible future applications in the field of behavioural neuroscience.

## Introduction

**Figure 1.**
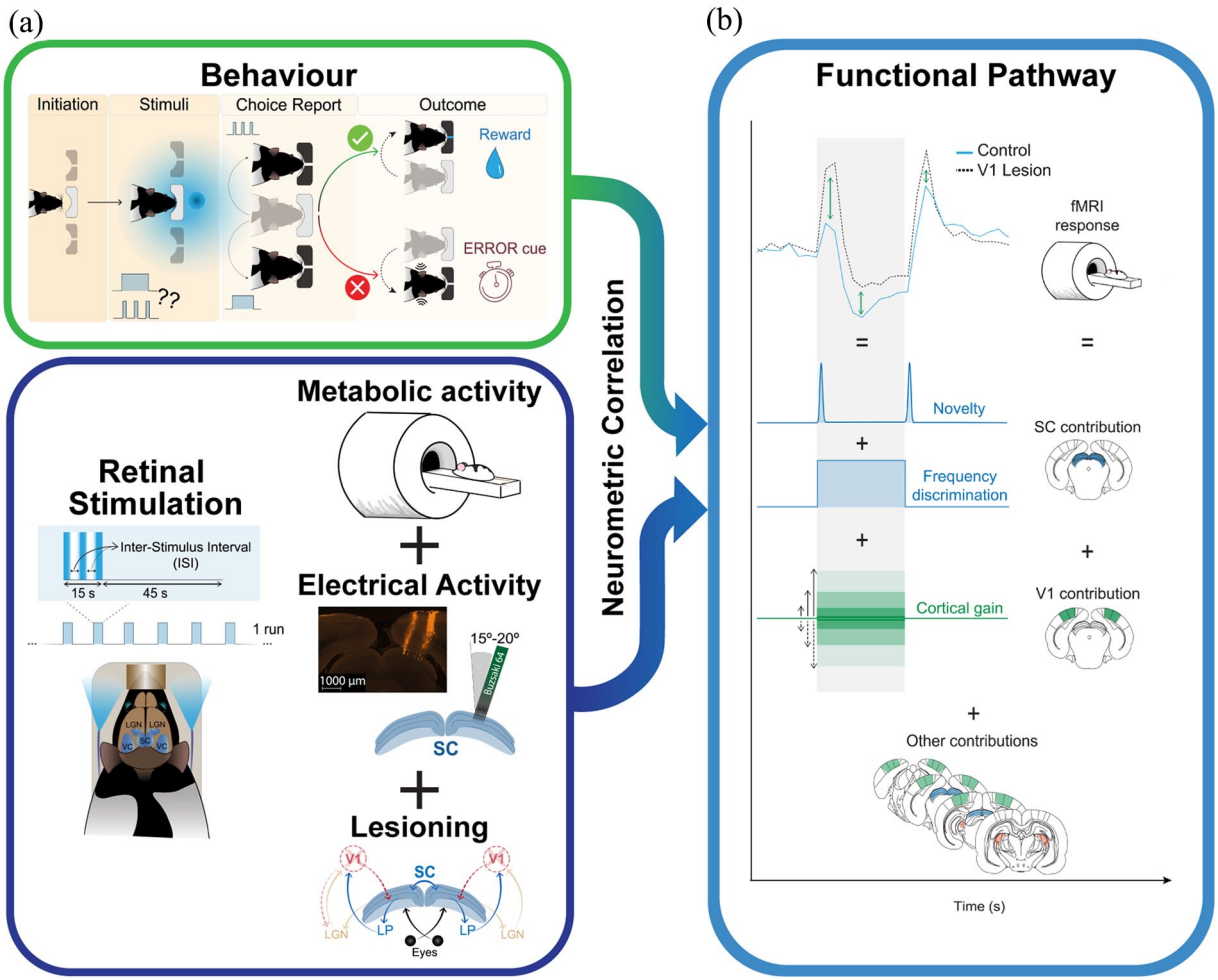
Graphical representation of the experimental strategy of Gil et al. (2024): (a) Multimodal behaviour, functional magnetic resonance imaging, electroencephalography, and causal intervention approaches to the investigation. (b) Compilation of approaches via neurometric correlation into a functional pathway outlining the contribution of the SC to cortical processing of the visual continuity illusion. Adapted with permission from Gil et al. (2024).

Distinguishing between static and dynamic visual stimuli is key in not only extracting information from the environment but also informing predators of the movement of prey and *vice versa*. The transition between static and dynamic visual states is known as the flicker fusion frequency (FFF) and refers to a phenomenon known as the visual continuity illusion (Mankowska et al., 2021). Here, a flashing light would appear solid due to the fusion of individual flashes. However, the mechanisms governing cortical responses at the transition between static and dynamic visual states are poorly understood. To shed light on this problem, Gil et al. (2024) used a multimodal approach involving behavioural assessment, functional magnetic resonance imaging (fMRI), electroencephalography (EEG) and targeted lesions to systematically investigate the mediators of visual temporal discrimination in the rat (Figure 1). Their study highlights the superior colliculus (SC) and subcortical visual pathways as the key substrates of visual temporal acuity by revealing neuronal and metabolic activation patterns that closely resemble behaviourally derived responses in a novel FFF operant task.

## Methods and findings

The behavioural task employed by Gil et al. (2024) was essentially a variation of a two-alternative forced-choice operant procedure. Water-restricted Long-Evans rats were trained to discriminate between either a continuous or flickering light-emitting diode (LED), reporting their choice in one of two apertures on either side of a central initiation port (Figure 1(a), top). Correct responses resulted in water reward, while incorrect responses were punished with a burst of white noise and a 5s time penalty. After training on an ‘easy’ discrimination (2 Hz) to criterion performance (>80% correct), animals were tested with 10 levels of increasing frequency ranging from 1 to 50 Hz relative to continuous light to establish individual FFF thresholds. High levels of accuracy were observed for frequencies less than 8 Hz, with a sharp drop-off in performance at higher frequencies. The data were modelled with a sigmoidal function and FFF threshold of 18 ± 2 Hz.

The authors next used fMRI in anaesthetised rats to identify changes in neuronal activity in early visual areas to steady-state LED stimulation (continuous, 1, 15, or 25 Hz; Figure 1(a), bottom). The findings revealed bidirectional, frequency-dependent, blood-oxygenation-level–dependent (BOLD) responses in visual cortex (VC) and SC. Compared to pre-stimulus baseline, high-frequency flashes resulted in negative BOLD responses (NBR) while low-frequency flashes produced positive BOLD responses (PBR). The SC also showed distinct ON/OFF PBR spikes as the stimulus frequency increased. However, correlational and neurometric mapping of frequency-dependent BOLD responses corresponded to SC activity only. Indeed, the resultant sigmoidal neurometric curve closely resembled the behaviourally derived FFF threshold of 19.8 ± 2.4 Hz. These findings suggest that neuronal activity is putatively suppressed at the transition from static to dynamic visual states and during dynamic vision and that this effect may be localised to the SC.

After identifying the SC as a key region for visual temporal processing, local field potentials (LFPs) were then recorded in the superficial SC (sSC) using steady-state LED stimulation in anaesthetised rats (Figure 1(a), bottom). Here, EEG spectrogram power traces revealed frequency-dependent patterning, with sub-behavioural FFF frequencies (1, 15 Hz) displaying distinct cyclic patterns and supra-behavioural FFF frequencies (25 Hz, continuous) showing steady-state responses. Notably, the bidirectional signals identified using fMRI were replicated in EEG power across all frequencies, suggesting an SC-mediated response to changes in the visual environment. In addition, mean multi-unit activity (MUA) power displayed a similar bidirectional response either side of the FFF as the BOLD responses, confirming a relationship between the two neural imaging techniques. EEG-derived behavioural correlation coefficients were significant, and neurometric curves also confirmed behaviourally relevant FFF thresholds for both the beginning (18 ± 1.7 Hz) and end (17.7 ± 2.4 Hz) of the steady-state stimulation, respectively.

Finally, Gil et al. (2024) irreversibly disrupted the primary VC (V1) using local infusions of ibotenic acid to abolish neural feedback from this region to the SC (Figure 1(a), bottom). The outcome of this intervention was greater ON/OFF peaks in BOLD response but reduced steady-state responses. Since the sSC still responded to visual stimuli in the absence of V1 feedback, the authors concluded that V1 may act as a gain regulator of SC responses to visual signals.

## Impact

Despite its importance for survival and perception, disparities are found in the reported FFFs of several species when measured cortically (Schwartz and Clark, 1957), behaviourally (Nomura et al., 2019) and from neuronal responses in the retina (Gilmour et al., 2008), leading to uncertainty both in the control and function of temporal discrimination mechanisms. Since retinal LFP and VC responses tend to overestimate and underestimate FFF thresholds, respectively (Wells et al., 2001), Gil and colleagues set out to test the hypothesis that the mechanism governing the true FFF threshold is mediated by a subcortical visual processing pathway. Their study provides, for the first time, convergent evidence establishing and validating the cortical control substrate of the visual continuity illusion. Their work suggests that the SC is the primary mediator of visual temporal discrimination, with novelty-based stimuli-independent ON/OFF signals followed by frequency-dependent steady-state responses and feedback response gain applied by V1 (Figure 1(b)). Key to this methodology is their novel behavioural task which accurately and reliably estimated the FFF in the Long-Evans rat and enabled the generation of neurometric curves for cross-modal comparison of behaviour with cortical recordings and interventions. Here, the behavioural FFF threshold was found to be markedly lower than previous estimates (Williams et al., 1985), a crucial observation for future experimental designs. The combination and synergy between the use of behaviour and quantitative neuroimaging and electrophysiology demonstrates how important convergent multimodal approaches are to mapping and validating previously disputed cortical pathways, not only for visual perception but also for other psychophysical processes as well.

## Discussion

Visual perceptual tasks are cognitively demanding with dynamic stimuli-focused states known to actively suppress attendance to other nearby stimuli (Yoo et al., 2022). This brings into question the use of anaesthetised animals for the present fMRI and EEG recordings, which may have misrepresented the role of V1 and other cortical regions involved in visual perception such as the posterior parietal cortex (Jia et al., 2020). Although, in the case of fMRI, sedation is likely unavoidable, incorporation of next-generation wireless electrocorticography coupled to behavioural performance and pharmacological intervention (Proctor et al., 2018; Xie et al., 2017) would be an important next step in understanding how task-engaged cortical responses contribute to the visual continuity illusion.

The authors acknowledged their study was not sufficiently powered to assess main or interactive effects of sex on behaviour and cortical responses. Hormonal changes during oestrus cycles may have impacted decision-making, and effort-based task models have shown oestradiol can modulate response preferences (Uban et al., 2012). Given the novelty of the present behavioural task, it would be important in future experiments to determine possible sex differences using a fully powered experimental design.

Overall, the work of Gil et al. (2024) provides compelling evidence supporting the SC as the subcortical mediator of temporal perceptual acuity in the rat. The study makes a significant contribution to a growing body of knowledge, validating multimodal methods as the future of cortical pathway interrogation, grounded firmly in rigorous behavioural assessment. The development of a broader repertoire of sophisticated interventions such as opto- and chemo-genetics alongside translationally relevant behavioural tasks is likely to accelerate our understanding of the cellular and subcellular mechanisms contributing to visual perception and other cognitive processes.
